# IL-17RA Signaling Reduces Inflammation and Mortality during *Trypanosoma cruzi* Infection by Recruiting Suppressive IL-10-Producing Neutrophils

**DOI:** 10.1371/journal.ppat.1002658

**Published:** 2012-04-26

**Authors:** Jimena Tosello Boari, María Carolina Amezcua Vesely, Daniela Andrea Bermejo, Maria Cecilia Ramello, Carolina Lucía Montes, Hugo Cejas, Adriana Gruppi, Eva Virginia Acosta Rodríguez

**Affiliations:** Centro de Investigaciones en Bioquímica Clínica e Inmunología (CIBICI-CONICET), Facultad de Ciencias Químicas, Universidad Nacional de Córdoba, Córdoba, Argentina; National Institute of Health, United States of America

## Abstract

Members of the IL-17 cytokine family play an important role in protection against pathogens through the induction of different effector mechanisms. We determined that IL-17A, IL-17E and IL-17F are produced during the acute phase of *T. cruzi* infection. Using IL-17RA knockout (KO) mice, we demonstrate that IL-17RA, the common receptor subunit for many IL-17 family members, is required for host resistance during *T. cruzi* infection. Furthermore, infected IL-17RA KO mice that lack of response to several IL-17 cytokines showed amplified inflammatory responses with exuberant IFN-γ and TNF production that promoted hepatic damage and mortality. Absence of IL-17RA during *T. cruzi* infection resulted in reduced CXCL1 and CXCL2 expression in spleen and liver and limited neutrophil recruitment. *T. cruzi*-stimulated neutrophils secreted IL-10 and showed an IL-10-dependent suppressive phenotype *in vitro* inhibiting T-cell proliferation and IFN-γ production. Specific depletion of Ly-6G+ neutrophils *in vivo* during *T. cruzi* infection raised parasitemia and serum IFN-γ concentration and resulted in increased liver pathology in WT mice and overwhelming wasting disease in IL-17RA KO mice. Adoptively transferred neutrophils were unable to migrate to tissues and to restore resistant phenotype in infected IL-17RA KO mice but migrated to spleen and liver of infected WT mice and downregulated IFN-γ production and increased survival in an IL-10 dependent manner. Our results underscore the role of IL-17RA in the modulation of IFN-γ-mediated inflammatory responses during infections and uncover a previously unrecognized regulatory mechanism that involves the IL-17RA-mediated recruitment of suppressive IL-10-producing neutrophils.

## Author Summary

IL-17 family is comprised for six members (IL-17A to F) that have been reported to play protective effects in bacterial and fungal infections and contradictory roles in parasite infections. Using mice deficient in IL-17RA, the common receptor subunit for many IL-17 family members, we determined that these cytokines are required for host protection against the parasite *Trypanosoma cruzi*. In absence of IL-17 signaling, mice developed an aggravated infection with similar levels of parasite in blood but increased inflammation and tissue damage of vital organs such as liver. We evaluated the mechanisms underlying this increased susceptibility and determined that the absence of IL-17RA caused a reduced arrival of neutrophils to organs such as spleen and liver. Neutrophils are phagocytic cells with abilities to directly destroy pathogens and also to regulate the inflammatory response. Indeed, we determined that neutrophils from *T. cruzi* infected mice are poisoned to secrete the regulatory cytokine IL-10. Finally, by experiments of depletion and adoptive transfer of neutrophils we determined that, during *T. cruzi* infection, IL-17RA is required for the recruitment of neutrophils that destroy the parasite and that also regulate inflammatory responses and collateral tissue damage by secreting IL-10.

## Introduction

The IL-17 cytokine family is formed by six members: IL-17A (also called IL-17), IL-17B, IL-17C, IL-17D, IL-17E (also called IL-25) and IL-17F [1]. The IL-17A and IL-17F are the best characterized members of the IL-17 family. These cytokines share the highest homology, are co-ordinately secreted by several subsets of immune cells [2] and can exist either as IL-17A and IL-17F homodimers or as IL-17A/IL-17F heterodimers [3]. Depending on the target cell population (epithelial and endothelial cells, fibroblasts, osteoblasts, and monocyte/macrophages), IL-17A and IL-17F induce the secretion of colony-stimulating factors (e.g., GM-CSF and G-CSF), CXC chemokines (e.g., CXCL1, CXCL2 and CXCL8), metalloproteinases, mucins, and proinflammatory cytokines (IL-6, IL-1 and TNF) [4]. Accordingly, IL-17A and IL-17F orchestrate a potent inflammatory response involving neutrophil recruitment and activation. In addition, these cytokines cooperate with TLR ligands, IL-1β and TNF to enhance inflammatory reactions and stimulate production of beta-defensins and other antimicrobial peptides [5]. Given these proinflammatory effects, production of IL-17A and IL-17F provides protection against a wide array of pathogenic microorganisms but also plays critical or contributing roles in several chronic inflammatory diseases [6].

Biological roles of IL-17B, IL-17C and IL-17D are less clear. IL-17B and IL-17C are able to stimulate the release of IL-1 and TNF from a human monocytic cell line and cause neutrophil infiltration [7], [8], whereas IL-17D induces expression of IL-6, IL-8 and GM-CSF from entothelial cells [9]. Furthermore, transfer of CD4+ T cells overexpressing IL-17B and IL-17C exacerbated collagen-induced arthritis [10]. Altogether, these antecedents suggest that IL-17B, IL-17C and IL-17D have similar activity to induce inflammatory mediators, and contribute to inflammatory responses like IL-17A and IL-17F [1]. In contrast, IL-17E has been reported to ameliorate chronic inflammatory diseases by suppressing Th1 and Th17 responses [11], [12], [13]. In addition, this cytokine, secreted by T cells, eosinophils and epithelial and endothelial cells, favors Th2 and Th9 responses and eosinophil recruitment [14]. Consequently, IL-17E plays protective roles against gastrointestinal helminth infections [15] but is deleterious in allergic settings [16].

The IL-17 receptor (IL-17R) family includes five members (IL-17RA to IL-17RE) that are thought to form homo or heterodimers to give raise to functional receptors [17]. For example, the receptor for IL-17A and IL-17F is a heterodimer formed by IL-17RA and IL-17RC [18], while the heterodimer consisting of IL-17RB and IL-17RA serves as receptor for IL-17E [19]. Recently, IL-17RA has been shown to interact with IL-17RE to form the signaling complex for IL-17C [20], [21]. Thus, IL-17RA would emerge as the common receptor chain for the IL-17 family whereas the other subunit of the receptor would provide specificity.

The protozoan parasite *Trypanosoma cruzi* is the causative agent of Chagas' disease, an endemic disease that affects 20 million people in Central and South America. In the last years, cases are more frequent in non-endemic areas in Europe and North America as consequence of people migration. Host resistance during experimental *T. cruzi* infection is dependent on both innate and acquired cell-mediated immune responses requiring the combined effects of many cell populations such as NK cells, CD4+, and CD8+ T cells [22]. Also antibodies are important for host survival and parasite clearance [23]. Concerning cytokines, IFN-γ plays a major protective role against *T. cruzi* infection by activating macrophages to destroy ingested parasites and to release proinflammatory cytokines [24], [25], [26]. Although required for parasite clearance, increased Th1-type response and high levels of IFN-γ and TNF have been associated to the pathogenesis of chronic Chagas disease [27], [28], [29], [30]. Furthermore, models of experimental *T. cruzi* infection using genetically-engineered mice such as WSX-1(IL-27R) deficient mice showed that a dysregulated proinflammatory cytokine production results in increased susceptibility to this parasite infection [31]. Indeed, deficient signaling of regulatory cytokines such as IL-10 correlated with increased mortality during experimental *T. cruzi* infection due to overwhelming inflammatory responses mediated by IFN-γ and TNF [32], [33]. Altogether, the literature supports the notion that during *T. cruzi* infection only a balanced production of inflammatory and anti-inflammatory factors will allow the control of parasite spreading without extensive collateral damage to the host.

The role of the IL-17 family during parasite infections is an emerging area of research with often contradicting results. In this regard, IL-17RA signaling has been shown to be both deleterious and protective during *Toxoplasma gondii* infection [34], [35]. Furthermore, two groups demonstrated that IL-17 plays a protective role in *T. cruzi* infection [36], [37], although the underlying mechanisms remains poorly understood. Indeed, both reports showed significant contradictions that might be related to the different experimental settings but deserves further investigation and discussion.

In this report, we determined that the three most studied members of the IL-17 family, IL-17A, IL-17E and IL-17F, are produced during *T. cruzi* infection. Furthermore, we confirmed that IL-17RA signaling is required for host resistance during the *T. cruzi* infection and focused at the mechanisms underlying IL-17RA-mediated protective effect. We determined that the signaling through this receptor is essential to regulate exaggerated Th1 inflammatory responses and the associated tissue damage by recruiting regulatory IL-10-producing neutrophils. Our results underscore the role of IL-17RA in the modulation of IFN-γ-mediated inflammatory responses during infections and uncover a previously unrecognized regulatory mechanism that likely involves IL-17 family-mediated recruitment of suppressive neutrophils.

## Results

### IL-17A, IL-17E and IL-17F are produced during T. cruzi infection and IL-17RA expression is required for host resistance

**Figure 1:**
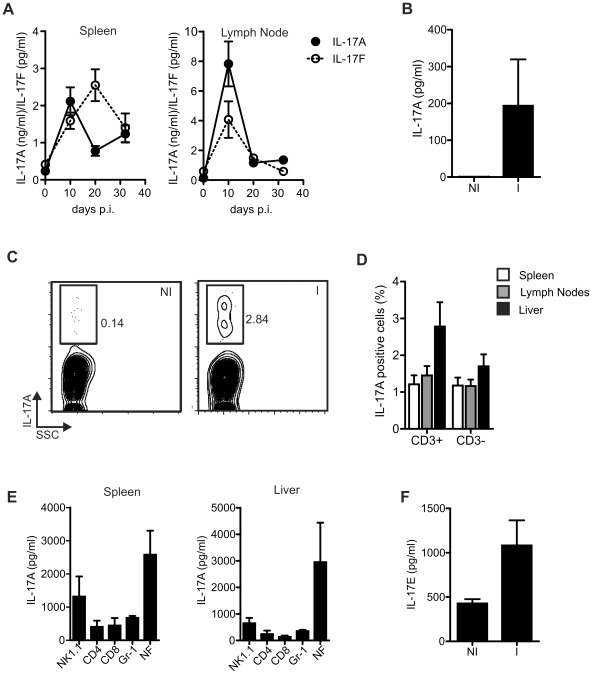
IL-17A, IL-17E and IL-17F production during *T. cruzi* infection. A) IL-17A and IL-17F concentration determined in the culture supernatants of spleen and lymph node cells obtained from mice at different times post *T. cruzi* infection and stimulated during 48 h in the presence of CD3/PdBU. Data are shown as mean ± SD, n = 5 mice per group. B) Plasma IL-17A concentration in non-infected (NI) or 20-day *T. cruzi*-infected (I) mice. Data are shown as mean ± SD, n = 5 mice per group. C) Percentage of IL-17A positive cells after 5 h PMA/Ionomycin stimulation of liver cell suspensions obtained from 20-day *T. cruzi*-infected mice. Plot representative of one out of five mice. D) Percentage of IL-17A positive cells within CD3 positive and CD3 negative cell population after 5 h PMA/Ionomycin stimulation of spleen, lymph nodes and liver cells from 20-day *T. cruzi*-infected mice. Data are shown as mean ± SEM, n = 5 mice per group. E) IL-17A concentration determined in the culture supernatants of NK1.1+ cells, CD4+ and CD8+ T cells, Gr-1+ cells and the remaining negative fraction (NF) sorted from the spleen and liver of 20-day *T. cruzi* infected mice and stimulated during 48 h. Data are shown as mean ± SD of triplicates cultures. F) Plasma IL-17E concentration in non-infected (NI) or 20-day *T. cruzi*-infected (I) mice. Data are shown as mean ± SD, n = 5 mice per group. Data in A, E and F and in B–D are representative of two and three independent experiments, respectively.

To evaluate the production of IL-17 cytokine family during the course of *T. cruzi* infection, we first focused in the best characterized inflammatory members IL-17A and IL-17F and quantified these cytokines in plasma and culture supernatants of cells obtained from C57BL/6 mice at different times post *T. cruzi* infection. Production of IL-17A and IL-17F by cells from the spleen and lymph nodes of infected mice peaked by days 10–20 post-infection and gradually decreased by day 32 post-infection (Figure 1A). Although detectable, IL-17F production was about 1000 times lower than IL-17A. Indeed, IL-17F was not quantifiable in plasma while IL-17A was detectable, but only during a short period around day 20 post *T. cruzi* infection (Figure 1B and data not shown). Moreover, in acutely infected mice IL-17A+ leukocytes were identified by flow cytometry in target organs such as the liver (Figure 1C). The IL-17A+ cells present in spleen, lymph nodes and liver of *T. cruzi* infected mice comprised both CD3+ as well as CD3− cells (Figure 1D). To further identify the cell sources of IL-17A during *T. cruzi* infection, different populations were sorted from spleen and liver of infected mice according to the expression of NK1.1, CD3, CD4, CD8 and Gr-1. Secretion of IL-17A was detected in the culture supernatants of NK cells, CD4+ and CD8+ T cells as well as Gr-1 (likely neutrophils) cells (Figure 1E). Furthermore, other populations were also able to secrete this cytokine as IL-17A production was detected in the culture supernatants of the negative fraction. Further experiments indicated that the other IL-17-producing populations comprised γδ T cells that have been reported as an important innate source of IL-17 in many infections [38] as well as another cell subset not previously described as IL-17 producer, which is matter of current research (data not shown). Next, we evaluated concentration of IL-17E, the member of the IL-17 family typified as anti-inflammatory cytokine. In contrast to IL-17A and IL-17F, IL-17E was readily detectable in the plasma of non-infected mice but its concentration was increased twice in the plasma of 20-day infected mice indicating that IL-17E was also induced during *T. cruzi* infection (Figure 1F).

**Figure 2:**
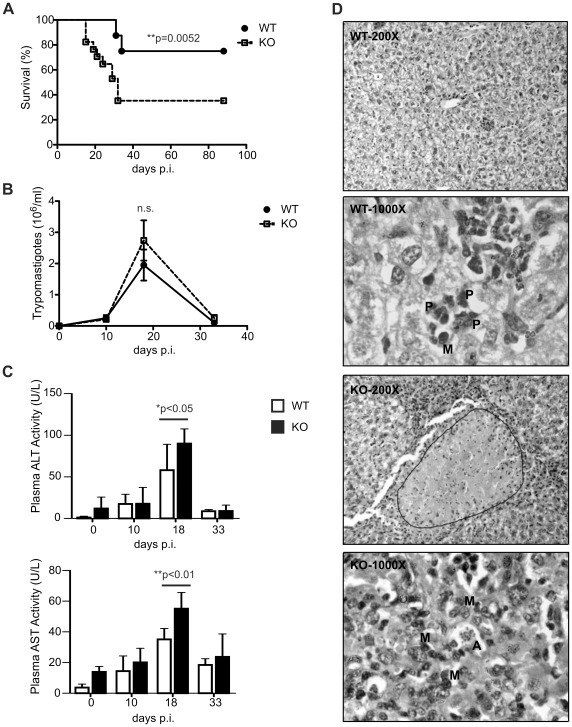
Increased mortality, tissue parasitism and hepatic damage in IL-17RA KO mice during *T. cruzi* infection. A) Survival of WT and IL-17RA KO mice after *T. cruzi* infection, P value calculated with a Gehan-Breslow-Wilcoxon test, n = 20 per group. B) Parasitemia determined at different time post *T. cruzi* infection in WT and IL-17RA KO mice, n = 10 per group. P values calculated using two-tailed T test. C) Plasma activity of ALT and AST in WT and IL-17RA KO mice at different times post *T. cruzi* infection. Data are shown as mean ± SD, n = 5 mice per group. P values calculated using two-way ANOVA followed by Bonferroni's posttest. D) Photographs of Hematoxilin/Eosin stained liver sections from 20-day *T. cruzi* infected WT and IL-17RA KO mice. 200× micrographs allow panoramic evaluation of the lesions. An extensive necrotic area is demarcated in the liver from KO mice. 1000× micrograph show details about the cellular alterations and the nature of inflammatory infiltrate (P: polymorphonuclear cells, M: mononuclear cells, A: amastigote nest). Photographs are representative of one out of five mice. Data in A–C and in D are representative of three and two independent experiments, respectively.

To address the role of IL-17 family during *T. cruzi* infection, IL-17RA KO mice were infected with *T. cruzi* and the progression of the infection was evaluated in comparison to wild-type (WT) mice. As illustrated in Figure 2A, IL-17RA KO mice showed increased mortality during *T. cruzi* infection. Because mortality during acute *T. cruzi* infection is often consequence of the combination between uncontrolled parasite replication and extended damage in vital organs; we evaluated these aspects in IL-17RA KO versus WT mice. We first determined that *T. cruzi*-infected IL-17RA KO mice showed similar parasitemia than WT controls (Figure 2B). To evaluate organ damage we focused in the liver and determined the plasma activity of the liver transaminases AST and ALT (Figure 2C). After 20 days of *T. cruzi* infection, increased AST and ALT activity were detected in WT and IL-17RA KO mice; however, the KO group presented significantly higher activity of the liver transaminases in plasma, suggesting increased liver damage. Histological analysis of the liver from acutely infected mice corroborated important evidences of liver injury in both WT and IL-17RA KO mice (Figures 2D and S1 in Text S1). Cellular infiltrate was already prominent by day 10 post-infection, augmented during the peak of the infection (day 18) and declined afterwards. Evidence of important cellular alterations was found during the peak of infection (day 18–20) and at later time points (day 32 post-infection). General hepatic structure was conserved but hepatocytes were round and swollen and showed vacuolar cytoplasm and picnotic nuclei. Kupffer's cell showed important hyperplasia. Focal necrosis and hyaline degeneration were observed in areas of varied extension depending on the group of mice evaluated. Of note, throughout the time course study and for all the evaluated parameters, but particularly in the extension of necrosis and hyaline degeneration, infected IL-17RA KO mice showed more severe lesions in comparison to WT mice (Table 1, Figure 2D and Figure S1 in Text S1). In addition, the predominant cell type present in the inflammatory infiltrates was clearly different between infected WT mice that showed neutrophilic infiltrate and infected IL-17RA KO mice that presented monocytic/lymphocytic infiltrate (Figure 2D and Table 1).

**Table 1:** Time course study of hepatic lesions in *T. cruzi* infected WT and IL-17RA KO mice.

Group (n = 5)	Day post-infection	Inflammatory infiltrate^a^ (main cell type)^b^	Necrosis^a^	Cellular lesions^a^
WT	10	+++ (PMN)	+	+
	18	++++ (PMN)	+	++
	32	+++ (PMN)	+	++
KO	10	++ (Mo/Ly)	+	++
	18	+++ (Mo/Ly)	++/+++	+++
	32	++ (Mo/Ly)	++/+++	+++

a Scale: +: mild; ++:moderate; +++: severe; ++++: extremely severe.

b PMN: Polymorphonuclear cells; Mo/Ly: monocyte/lymphocytes.

### Increased IFN-γ production in T. cruzi-infected IL-17RA KO mice correlates with hepatic damage and mortality

**Figure 3:**
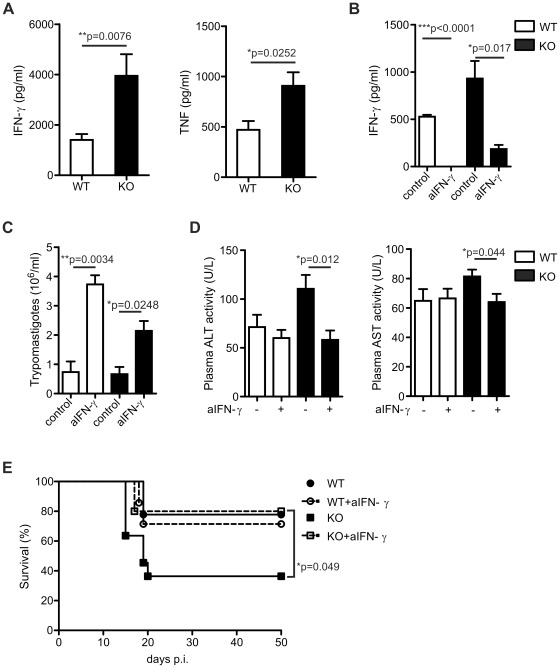
Augmented production of IFN-γ caused increased hepatic damage and mortality in *T. cruzi* infected IL-17RA KO mice. A) Plasma IFN-γ and TNF concentration in 20-day *T. cruzi* infected WT and IL-17RA KO mice. Data are shown as mean ± SD, n = 6 mice per group. P values calculated using two-tailed T test. B) Plasma IFN-γ concentration in 20-day *T. cruzi* infected WT and IL-17RA KO mice treated with anti-IFN-γ. P values calculated using two-tailed T test. C) Parasitemia determined in 20-day *T. cruzi* infected WT and IL-17RA KO mice treated with anti-IFN-γ. Data are shown as mean ± SD, n = 6 mice per group. P values calculated using two-tailed T test. D) Activity of ALT and AST determined in the plasma of 20-day *T. cruzi* infected WT and IL-17RA KO mice treated with anti-IFN-γ. Data are shown as mean ± SD, n = 6 mice per group. P values calculated using two-tailed T test. E) Survival of *T. cruzi* infected WT and IL-17RA KO mice treated with anti-IFN-γ. P value calculated with a Gehan-Breslow-Wilcoxon test, n = 12 per group. Data in A and in B–E are representative of four and two independent experiments, respectively.

Because dysregulated inflammatory responses can contribute to organ damage and mortality during *T. cruzi* infection, we evaluated the levels of proinflammatory cytokines such as IFN-γ and TNF in the serum of *T. cruzi* infected WT and IL-17RA KO mice. At day 20 post-infection, plasma levels of both cytokines were significantly higher in IL-17RA KO mice than in WT mice (Figure 3A). Accordingly, 20-day infected IL-17RA KO mice showed increased percentage of IFN-γ+ T cells in spleen and liver in comparison to WT control (Figure S2A in Text S1). Moreover, spleen CD4+ and liver CD8+ T cells and spleen and liver CD8+ T cells from infected IL-17RA KO mice secreted more IFN-γ and TNF, respectively, than WT counterparts (Figure S2B in Text S1).

To address the role of the augmented IFN-γ response in the increased susceptibility of IL-17RA KO mice to *T. cruzi*, we evaluated the progression of the infection in mice receiving a IFN-γ blocking treatment. Injection of neutralizing anti-IFN-γ monoclonal antibodies (Abs) greatly reduced plasma IFN-γ concentration in *T. cruzi* infected WT and IL-17RA KO mice (Figure 3B). As expected due to the role of this cytokine in parasite control, IFN-γ blockade during *T. cruzi* infection resulted in higher parasitemia in WT and IL-17RA KO mice after 20 days post-infection (Figure 3C). In contrast, the plasma activity of the hepatic transaminases ALT and AST as well as histopathological damage of the liver were significantly diminished in infected IL-17RA KO but not in infected WT mice after the anti-IFN-γ treatment (Figure 3D and Table 2). Overall, the blocking treatment did not affect the survival of infected WT mice but increased survival of infected IL-17RA KO mice to levels comparable to the WT control group (Figure 3E).

**Table 2:** Hepatic lesions in anti-IFN-γ treated *T. cruzi* infected WT and IL-17RA KO mice at day 20 post-infection.

Group (n = 5)	Inflammatory infiltrate^a^ (main cell type)^b^	Necrosis^a^	Cellular lesions^a^
WT Control	+++ (PMN)	++	+++
WT anti-IFN-γ	++++ (PMN)	+++	+++
KO Control	++/+++ (Mo/Ly)	++++	++++
KO anti-IFN-γ	+++ (Mo/Ly)	+	+++

a Scale: +: mild; ++:moderate; +++: severe; ++++: extremely severe.

b PMN: Polymorphonuclear cells; Mo/Ly: monocyte/lymphocytes.

### Neutrophil recruitment is reduced in T. cruzi infected IL-17RA KO mice

**Figure 4:**
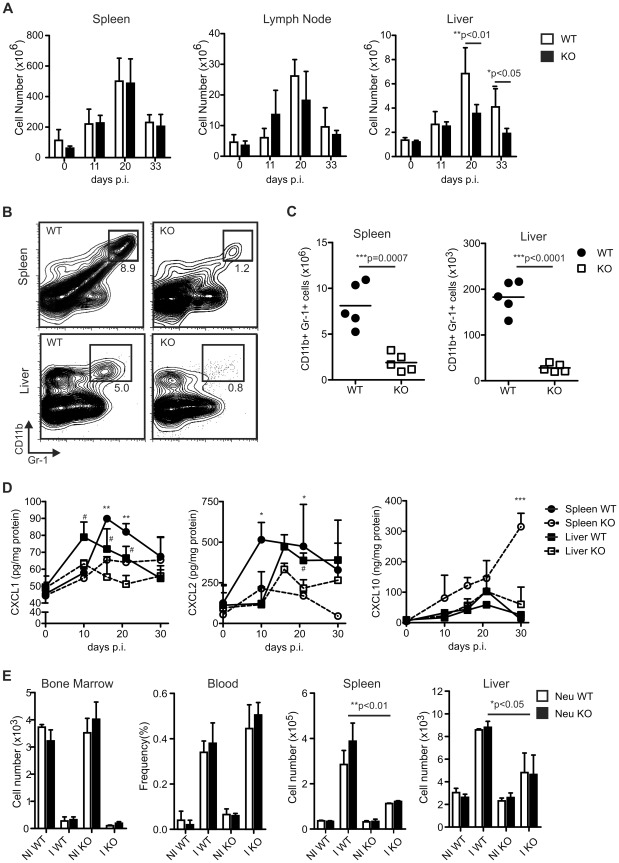
Reduced neutrophil chemoattractant production and neutrophil recruitment in the spleen and liver of *T. cruzi* infected IL-17RA KO mice. A) Cell numbers in spleen, lymph nodes and liver of WT and IL-17RA KO mice determined at different times after *T. cruzi* infection. Data are shown as mean ± SD, n = 5 mice per group. P values calculated using two-way ANOVA followed by Bonferroni's posttest. B) Percentage of CD11b+ Gr-1+ neutrophils in spleen and liver of 20-day *T. cruzi* infected WT and IL-17RA KO mice. Plots are representative one out of five mice. C) Absolute numbers of CD11b+ Gr-1+ neutrophils in spleen (left) and liver (right) of 20-day *T. cruzi* infected WT and IL-17RA KO mice. Each symbol represents a different mouse and horizontal line indicates the mean. P values calculated with two-tailed T test. D) Concentration of CXCL1, CXCL2 and CXCL10 in spleen and liver homogenates obtained from WT and IL-17RA KO mice at different times post *T. cruzi* infection. Data are shown as mean ± SD of biological triplicates, normalized to total protein concentration, n = 5 mice per group. P values calculated with two-way ANOVA followed by Bonferroni's posttest. (* p: spleen WT vs spleen KO; # p: liver WT vs liver KO). E) Absolute number or frequency of transferred WT and IL-17RA KO neutrophils detected in bone marrow, blood, spleen and liver of non-infected (NI) and infected (I) WT and IL-17RA KO mice 3 h after i.v. injection. Data are shown as mean ± SD, n = 5 per group. P values calculated with two-tailed T test. Data in A–C and D–E are representative of four and two independent experiments, respectively.

We next sought to determine whether the dysregulated IFN-γ response observed in infected IL-17RA KO mice correlated with an altered expansion or distribution of immune cell populations in secondary lymphoid or target organs. To this end, we first compared the total cell numbers in spleen, lymph nodes and liver from infected WT and IL-17RA KO mice. During the course of the infection, both group of mice presented similar cell numbers in secondary lymphoid organs, but infected IL-17RA KO mice showed a reduced number of infiltrating cells in liver (Figure 4A). In particular, we found that, in comparison to WT controls, *T. cruzi* infected IL-17RA KO mice presented a significant reduction in the frequency and absolute numbers of a CD11b+Gr-1+cell population in spleen and liver (Figure 4B and 4C). Evaluation of the expression of several surface markers in the CD11b+Gr-1+ population showed a pattern compatible with neutrophils (i.e. Ly-6G^high^; Ly-6C+ and F4/80− with variable expression of CD11c according to the organ source) (Figure S3A in Text S1). Neutrophil morphology was confirmed by optic microscopy (Figure S3B in Text S1).

Reduced neutrophil numbers in spleen and liver of *T. cruzi* infected IL-17RA KO mice was unlikely consequence of a deficient myelopoiesis because, similar to infected WT animals, these mice presented increased percentages and absolute numbers of neutrophils in bone marrow and blood in comparison to non-infected controls (Figures S4A and B in Text S1). Accordingly, *T. cruzi* infected IL-17RA KO and WT mice showed no differences in the concentration of the neutrophil growth factor G-CSF in spleen and liver (Figure S4C in Text S1). Next, we evaluated the production of chemokines such as CXCL1 (KC) and CXCL2 (MIP-2), known to act as neutrophil chemoattractants [39], [40] and to be modulated by the IL-17 family [41]. We determined that both chemokines were clearly induced by *T. cruzi* infection in the spleen and liver of WT mice but, throughout the infection period evaluated or at least during the peak of the infection (day 20 post-infection), showed reduced concentration in the same tissues from IL-17RA KO mice (Figure 4D). In contrast, the concentration of CXCL-10 (IP-10), a chemokine induced by IFN-γ [42], showed a tendency to be higher in the spleen and liver of *T. cruzi* infected IL-17RA KO mice in comparison to WT controls although the increase was statistically significant only in the spleen after 30 days post-infection (Figure 4D). Altogether these results suggested that the absence of IL-17RA signaling during *T. cruzi* infection affected the recruitment rather than the development or survival of neutrophils. Similar impairment in neutrophil recruitment was reported in other experimental infections using mice deficient for IL-17 signaling [35], [43].

To confirm the impairment in neutrophil recruitment, we injected dye-stained neutrophils purified from bone marrow of WT and IL-17RA KO mice and evaluated their distribution/migration in infected and non-infected mice (Figure 4E). Three hours after intravenous injection, neutrophils were mainly present in the bone marrow of non-infected WT and IL-17RA KO mice. The distribution of the injected cells was different in *T. cruzi* infected mice with low number of the transferred neutrophils detected in the bone marrow and preferential presence in blood and organs. Thus, infected WT and IL-17RA KO mice presented similar numbers of the injected Ly-6G+ neutrophils in the blood and bone marrow. Furthermore, injected neutrophils were recruited into the spleen and liver of infected WT mice, independently on the expression of IL-17RA on the injected neutrophils themselves. In contrast, but in concordance with their lower production of CXCL1/CXCL2, significantly lesser numbers of the injected neutrophils reached spleen and liver of infected IL-17RA KO mice compared to infected WT controls. These results confirmed that lack of IL-17RA in host cells, but not in the migrating neutrophils themselves, were required for proper neutrophil recruitment into tissues during *T. cruzi* infection.

### T. cruzi infection promotes the differentiation of IL-10 producing neutrophils that show suppressive function in vitro

**Figure 5:**
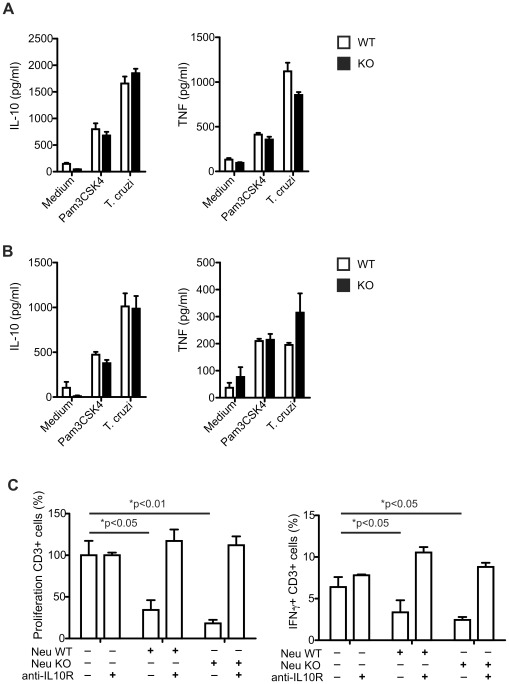
Suppression of T cell proliferation and IFN-γ production by IL-10 secreting neutrophils obtained from *T. cruzi* infected mice. A–B) Concentration of IL-10 and TNF determined in 48 h culture supernatants of Ly-6G+ neutrophils purified from bone marrow of WT and IL-17RA KO mice (A) and of CD11b+Ly-6G+ neutrophils sorted from spleen 20-day *T. cruzi* infected WT and IL-17RA KO mice (B) and stimulated as indicated. Data are shown as mean ± SD of cuatriplicates. C) Proliferation and percentage of IFN-γ-producing CD3 positive splenocytes from normal mice after 5 day stimulation in anti-CD3 and anti-CD28 coated plates in the presence of CD11b+Ly-6G+ neutrophils obtained from 20-day *T. cruzi* infected WT and IL-17RA KO mice and of a blocking anti-IL10R Ab. Data are shown as mean ± SD of triplicates. P values calculated using two-tailed T test. Data in A–C are representative of two independent experiments.

Besides their innate role as phagocytes, neutrophils interact with other immune cells and secrete cytokines able to regulate adaptive cellular immune responses [44]. To elucidate whether reduced neutrophil recruitment during *T. cruzi* infection may affect not only direct parasite control but also IFN-γ secretion, we analyzed neutrophil cytokine production in our experimental settings. Neutrophils purified from bone marrow of non-infected WT and IL-17RA KO mice and cultured with live *T. cruzi* tripomastigotes secreted IL-10 and TNF, but minimal or no IL-1β, IL-6 and IL-12p70 (Figure 5A, Figure S5 in Text S1 and data not shown). Furthermore, CD11b+Ly-6G+ neutrophils sorted from the spleen of *T. cruzi* infected WT and IL-17RA KO mice and stimulated with live *T. cruzi* produced higher levels of IL-10 and similar levels of TNF compared to Pam3CSK4 stimulated neutrophils (Figure 5B). To evaluate the possible regulatory effect of IL-10-secreting neutrophils, we performed a typical *in vitro* suppression experiment determining cell proliferation and cytokine production of CFSE-labeled splenocytes activated with anti-CD3 and anti-CD28 in the presence or absence of neutrophils. CD3+ splenocytes cultured in the presence of spleen neutrophils sorted from infected WT and IL-17RA KO mice showed reduced proliferation and frequency of IFN-γ producing cells. Blockade of IL-10R in these co-cultures restored CD3+ splenocyte proliferation and IFN-γ production (Figure 5C).

### Neutrophils regulates IFN-γ production and hepatic damage in vivo during T. cruzi infection

**Figure 6:**
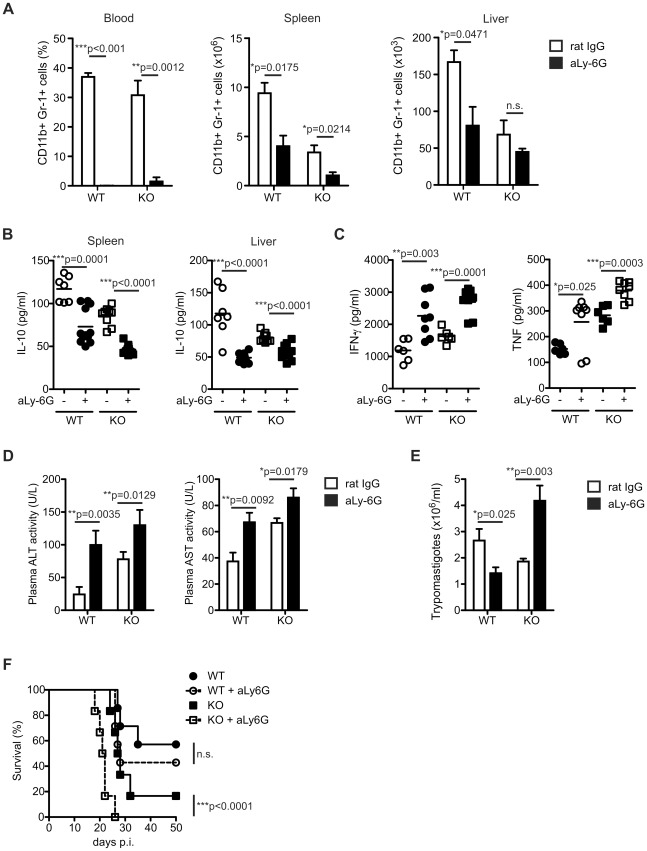
Increased IFN-γ production and hepatic transaminases activity in *T. cruzi* infected WT and IL-17RA KO mice after neutrophil depletion. A) CD11b+Gr-1+ frequency and absolute numbers in blood, spleen and liver of 20-day *T. cruzi* infected WT and IL-17RA KO mice treated with anti-Ly6G mAbs. Data are shown as mean ± SD, n = 6–8 mice per group. P values calculated by two-tailed T test. B) Concentration of IL-10 determined in 48 h unstimulated culture supernatants of spleen and liver cell suspensions obtained from 20-day *T. cruzi* infected WT and IL-17RA KO mice treated with anti-Ly-6G mAbs. Each symbol represents a different mouse and horizontal line indicates the mean. P values calculated with two-tailed T test. C) Plasma IFN-γ and TNF concentration in 20-day *T. cruzi* infected WT and IL-17RA KO mice treated with anti-Ly-6G mAbs. Each symbol represents a different mouse and horizontal line indicates the mean. P values calculated with two-tailed T test. D) Activity of ALT and AST determined in the plasma of 20-day *T. cruzi* infected WT and IL-17RA KO mice treated with anti-Ly-6G. Data are shown as mean ± SD, n = 6–8 mice per group. P values calculated using two-tailed T test. E) Parasitemia determined in 20-day *T. cruzi* infected WT and IL-17RA KO mice treated with anti-Ly-6G Abs. Data are shown as mean ± SD, n = 6–8 mice per group. P values calculated using two-tailed T test. F) Survival of *T. cruzi* infected WT and IL-17RA KO mice treated with anti-Ly-6G. P value calculated with a Gehan-Breslow-Wilcoxon test, n = 12 per group. Data in A–D and in E are representative of three and two independent experiments, respectively.

To understand the *in vivo* biological relevance for *T. cruzi* infection of the reduced neutrophil numbers observed in the liver and spleen of IL-17RA KO mice, we performed neutrophil depletion experiments using an anti-Ly-6G specific monoclonal Ab. Our injection scheme completely depleted CD11b+Ly-6G+ neutrophils from blood of *T. cruzi* infected WT and IL-17RA KO mice and reduced the number of these cells in organs such as spleen and liver (Figure 6A). Neutrophil depletion significantly reduced IL-10 secretion by spleen and liver cell suspensions from 20-day infected WT and IL-17RA KO mice (Figure 6B), indicating that these cells are an important source of IL-10 during the acute phase of *T. cruzi* infection. Likely as a consequence of the reduced IL-10 levels, anti-Ly-6G treatment raised the plasma concentration of IFN-γ and TNF in *T. cruzi* infected WT and IL-17RA KO mice (Figure 6C). Of note, the increased levels of type 1 proinflammatory cytokines observed in both neutrophil-depleted groups of mice correlated with increased liver damage as determined by the significant augment in the plasma activity of hepatic transaminases (Figure 6D). Regarding parasitemia, the anti-Ly-6G treatment had opposite outcomes according to the group of mice and resulted in lower and higher blood parasite numbers in infected WT and IL-17R KO mice, respectively (Figure 6E). This discrepant result would reflect how differently an enhanced IFN-γ mediated inflammatory response affected infection progression in each group of mice. Thus, increased IFN-γ levels in neutrophil depleted WT mice favored liver damage but also facilitated parasite control. In contrast, even higher levels of inflammatory cytokines in anti-Ly-6G treated IL-17RA KO mice induced devastating tissue damage that likely lead to the inability to control parasite replication. Indeed, neutrophil depletion significantly augmented mortality and wasting disease in infected IL-17R KO mice and showed a tendency to reduce survival in infected WT mice although it was not statistically significant when compared to the control group (Figure 6F).

**Figure 7:**
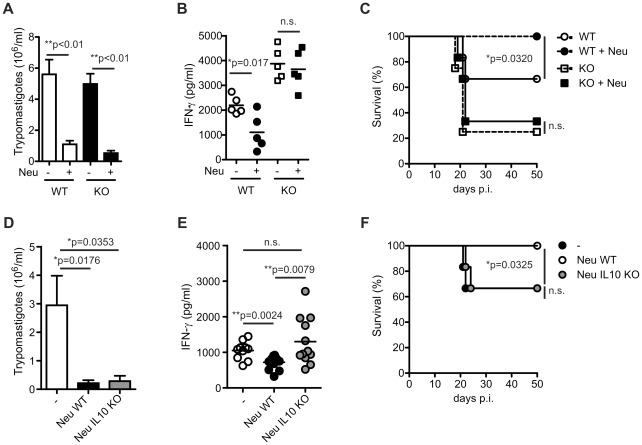
IL-10-dependent modulation of IFN-γ production by adoptively transferred neutrophils during *T. cruzi* infection. A) Parasitemia determined at day 20 post-infection in infected WT and IL-17RA KO mice adoptively transferred with WT neutrophils. Data are shown as mean ± SD, n = 6 per group. P values calculated with two-tailed T test. B) Concentration of IFN-γ in plasma of 20-day *T. cruzi* infected WT and IL-17R KO mice adoptively transferred with WT neutrophils. Data are shown as mean ± SD, n = 6 per group. P values calculated with two-tailed T test. C) Survival of *T. cruzi* infected WT and IL-17R KO mice adoptively transferred with WT neutrophils. P value calculated with a Gehan-Breslow-Wilcoxon test, n = 12 per group. D) Parasitemia determined at day 20 postinfection in infected WT mice adoptively transferred with WT and IL-10 KO neutrophils. Data are shown as mean ± SD, n = 12 per group. P values calculated with two-tailed T test. E) Concentration of IFN-γ in plasma of 20-day *T. cruzi* infected WT mice adoptively transferred with WT and IL-10 KO neutrophils. Data are shown as mean ± SD, n = 12 per group. P values calculated with two-tailed T test. F) Survival of *T. cruzi* infected WT mice adoptively transferred with WT and IL-10 KO neutrophils. P value calculated with a Gehan-Breslow-Wilcoxon test, n = 12 per group. Data in A–F are representative of two independent experiments.

As a second approach to evaluate the role of neutrophil during the course of *T. cruzi* infection, we performed adoptive transfer experiments with a scheme involving four injections of WT neutrophils at different times post-infection. Neutrophil adoptive transfer resulted in a significant drop in the parasitemia levels in both infected WT and IL-17RA KO mice (Figure 7A). This would reflect the ability of the injected neutrophils to directly kill parasites independent on migration into peripheral tissues. In contrast, and likely as consequence of differences in the tissue distribution of the transferred neutrophils between infected WT and IL-17RA KO mice (Figure 4E), neutrophil injection diminished plasma IFN-γ concentration only in infected WT mice (Figure 7B). In correlation with the combined reduction in blood parasite numbers and proinflammatory IFN-γ production, neutrophil-injected infected WT but not IL-17RA KO mice showed increased survival in comparison to controls (Figure 7C). To further elucidate the role of the IL-10 produced by the injected neutrophils in the regulation of IFN-γ production, we repeated the adoptive transfer experiment with neutrophils purified from WT and IL-10-deficient mice using infected WT mice as recipients. Lack of IL-10 production by the injected neutrophils did not affect their ability to reduce blood parasite numbers (Figure 7D) but, in agreement with our *in vitro* results (Figure 5C), significantly impaired their capacity to decreased plasma IFN-γ concentration (Figure 7E). Furthermore, in contrast to WT neutrophils, adoptive transfer of IL-10 deficient neutrophils failed to reduce the mortality of the infected WT recipient (Figure 7F).

## Discussion

Several members of the IL-17 family have been reported to have protective roles in host resistance against different microbes [1]. Such a role for IL-17A, the founding member of this family, as well as for IL-17F during bacterial and fungal infections has been largely recognized [45]. Furthermore, IL-17C has been recently shown to trigger similar effector responses than IL-17A and to participate in the immunity against certain intestinal bacteria [21]. Also IL-17E contributes to host resistance against gut microorganisms leading to the control of helminth infections by inducing Th2 responses [46]. Although increasing evidence supports a protective role for IL-17 family in extracellular infections, its function during infections with intracellular microorganisms where Th1 are required for host resistance remains less clear. Particularly during *T. cruzi* infection, IL-17A was reported to play a protective role by two independent groups [36], [37]; however, both reports showed important contradictions and the underlying mechanisms remained unresolved. Moreover, both studies used different experimental approaches that targeted IL-17A but not other IL-17 cytokines such as IL-17F and IL-17C that would have similar effector functions and could compensate for the lack of IL-17A. Indeed, we determined that during *T. cruzi* infection not only IL-17A but also IL-17E and IL-17F are produced during the peak of the infection. Whether other cytokines of the IL-17 family are induced by *T. cruzi* infection and the signals involved in their induction as well as the identity of cytokine-producing cell subsets are matter of current research in our laboratory.

Using IL-17RA deficient mice allowed us to understand the contribution of the IL-17 family to host resistance during *T. cruzi* infection overcoming the possible redundant function of some of its members. Indeed, our results differ in some aspects with those of *Miyazaki et al* that used IL-17A KO mice that specifically lacked IL-17A but had conserved signaling for the other members of the family. In contrast, our findings are more similar to those of da Matta Guedes *et al* that used a blocking Ab to neutralize IL-17A that could be crossreacting with other members highly homologous to IL-17A such as IL-17F, inhibiting all their functions. In this context, we determined that the absence of IL-17RA expression lead to increased mortality during the acute phase of *T. cruzi* infection. Increased mortality was not associated to augmented parasitemia but rather correlated with an exaggerated inflammatory response that severely affected liver and likely other vital organs such as heart and kidneys as described for IL-17−/− mice [37]. Indeed, we formally demonstrated that the susceptible phenotype of IL-17RA KO mice to *T. cruzi* infection was, at least in part, a consequence of a dysregulated inflammatory response caused by an exacerbated production of type 1 inflammatory cytokines such as IFN-γ and TNF production. Thus, IFN-γ blockade in *T. cruzi* infected IL-17RA KO mice mimicked the more resistant phenotype (increased survival and moderate liver damage) observed in WT mice.

From the cytokines that signals through IL-17RA, the proinflammatory IL-17A was surprisingly reported to suppress pathological IFN-γ dependent inflammation preventing tissue destruction [47]. Thus, in a model of graft-versus-host disease, IL-17A deficiency elicited an amplified Th1 response with high IFN-γ levels and greater severity of the disease [48]. Furthermore, IL-17A prevented and IL-17F exacerbated IFN-γ production and tissue destruction in a dextran sodium sulfate colitis model [49]. Also IL-17E, early classified as an anti-inflammatory cytokine, is able to inhibit IFN-γ as well as IL-17 production during infections and autoimmune settings [50]. A contrasting scenario was depicted for the last cytokine described to use IL-17RA as receptor complex, as the few available studies point at a proinflammatory role for IL-17C through the induction of IL-17A but not IFN-γ [20]. Thus, considering the available literature, it is likely that the phenotype of exuberant IFN-γ production, tissue damage and mortality observed in *T. cruzi* infected IL-17RA deficient mice could be attributed to the lack of response to not only IL-17A and IL-17F but also IL-17E. Furthermore, we cannot rule out that also the lack of response to IL-17C may somehow contribute to the susceptible phenotype of IL-17RA KO mice during *T. cruzi* infection. Further studies using mice deficient for individual or combined cytokines will be required to understand the specific contribution of each IL-17 family member.

Besides the exacerbated type 1 inflammatory responses, lack of IL-17RA expression during *T. cruzi* infection resulted in decreased neutrophil numbers in organs such as spleen and liver that kinetically correlated with impaired production of the neutrophil chemoattractants CXCL1 and CXCL2 in those peripheral tissues. Although some chemokines involved in neutrophil migration [51] may not be affected by IL-17 family and could account for the few neutrophils present in infected IL-17RA KO mice, the reduction in CXCL1/CXCL2 has been proved to greatly affect neutrophil recruitment into tissues in many inflammatory conditions [51] and likely explains the deficient neutrophil migration in infected IL-17RA KO mice. These results focused our work at investigating the relationship that linked the IL-17RA-induced neutrophil recruitment with the inflammation during *T. cruzi* infection. Neutrophils were previously reported to play either protective or deleterious roles during Chagas' disease according to the mice strain [52]. However, this conclusion was based in the depletion of Gr-1+ cells that has been shown to comprise not only neutrophils but also dendritic cells, monocytes, macrophages and lymphocytes and therefore, the reported effects cannot be solely ascribed to neutrophils [53]. Consequently, the specific role of neutrophils during *T. cruzi* infection remained elusive and should be reevaluated using newly available and highly specific tools such as the specific Ly-6G (1A8) mAbs.

Even though neutrophils were historically regarded as strict innate cells characterized by unspecific killing abilities and proinflammatory properties, accumulating data suggest that these cells express a vast array of pattern recognition receptors and respond to environmental cues producing several cytokines and chemokines that modulate innate and adaptive immunity [44]. Indeed, the crosstalk between neutrophils and T cells has been described in many physiological and pathological conditions, including acute and chronic inflammation during infections and cancer [54]. Noteworthy, adoptive transfer experiments as well as specific Ly-6G+ neutrophil depletion allowed us to uncover the critical and unexpected role of neutrophil in the regulation of type 1 inflammatory response and tissue damage during *T. cruzi* infection. Thus, repetitive adoptive transfer of neutrophils during *T. cruzi* infection resulted in lower parasitemia in both WT and IL-17RA KO mice and in reduced IFN-γ production only in WT mice. These findings may reflect that neutrophils could be directly involved in the killing or phagocytosis of circulating *T. cruzi* as reported for blood-stage plasmodium [55]. Therefore, presence of additional neutrophils in the blood of WT and IL-17RA KO mice may help to lower the parasitemia in a mechanism rather independent on migration into tissues or IFN-γ levels. In contrast, neutrophil regulation of the adaptive response (i.e. downregulation of IFN-γ production) was restricted to WT mice as it would require the IL-17-dependent recruitment of the injected neutrophil to immune induction or effector sites. Interestingly, the reduction of parasitemia levels after neutrophil transfer was not enough to prevent mortality as neutrophil-injected IL-17RA KO mice showed the same mortality than the control KO group. In contrast, the decrease of the IFN-γ levels and the associated inflammatory liver damage together the lower parasitemia would account for the increased survival of infected WT mice injected with neutrophils.

In the same direction, an anti-Ly6G treatment resulted in the amplification of IFN-γ and TNF production in infected WT and IL-17R KO mice likely as consequence of reduced production of the regulatory cytokine IL-10 after neutrophil depletion. The augmented type 1 inflammatory response in infected WT mice correlated with increased plasma levels of the hepatic transaminases but also with reduced parasitemia and, in overall, did not significantly affect survival. Thus, although anti-Ly6G-treated infected WT mice resembled infected IL-17RA KO mice in some aspects such as exuberant IFN-γ and TNF production and enhanced liver damage, they did not show the same high mortality rate. There are several possible and not mutually exclusive causes for such difference. One possible explanation is that additional mechanisms besides the reduced neutrophil numbers are involved in the exacerbated inflammatory response and reduced resistance to *T. cruzi* infection of IL-17RA KO mice. In this regard, we are evaluating the possible contribution of IL-17E in the recruitment of eosinophils and the suppression inflammatory responses. Moreover, T-cell intrinsic effect are not ruled out and as suggested in a colitis model [49], lack of IL-17RA signaling may release IL-17 mediated suppression of T-bet expression facilitating an aberrant Th1 differentiation program independent on other extrinsic signals. In addition, IL-17A has been reported to be required for the development of cytotoxic and humoral responses [56], [57], therefore parasite-specific adaptive immune responses may be undermined in *T. cruzi* infected mice lacking IL-17RA signaling contributing to reduced resistance to infection. Finally, another possible explanation is that the few neutrophils that remained in tissues of anti-Ly6G-treated WT infected mice may be able to partially contain overwhelming inflammation and organ injury. In this regard, the depleting treatment in infected IL-17RA KO mice that *per se* presented reduced tissue neutrophil numbers further exacerbated the already exuberant IFN-γ and TNF secretion and worsened liver damage, resulting in wasting disease, higher parasitemia and increased mortality. Altogether, these results support the notion that during *T. cruzi* infection, besides their innate function in parasite control, neutrophils play a regulatory role on the adaptive immune response with beneficial and detrimental effects in host resistance. Of note, during *T. cruzi* infection Foxp3+ regulatory T cells have been reported to play limited or even irrelevant roles [58], [59]. Therefore, in a context where the main regulatory cell subset showed reduced relevance, other cell populations with some regulatory abilities (i.e. neutrophils, regulatory B cells, M2 macrophages, etc) may take over the regulatory stage.

The suppressive role of neutrophils has been recognized in the recent years, mainly through the description of myeloid suppressor cells [60]. In addition, two recent reports described the existence of suppressor IL-10-producing neutrophils during a bacterial infection and melanoma [61], [62]. Furthermore, a recent work using IL-10-β-lactamase reporter mouse revealed that neutrophils are the major source of IL-10 during systemic *Yersinia enterocolitica* infection [63]. In this context, we demonstrated that bone marrow neutrophils stimulated with live *T. cruzi* produced IL-10 as well as TNF but not other proinflammatory cytokines. Furthermore, spleen neutrophils purified from infected mice produced high levels of IL-10 and low levels of TNF after restimulation with the parasite suggesting that *T. cruzi* infection somehow poisoned a “regulatory” status in these cells. Remarkably, neutrophils purified from the spleen of *T. cruzi* infected mice showed a suppressor function as they inhibited T cell proliferation and IFN-γ production in an IL-10R-dependent manner. Dependency on IL-10 for the suppressive effect of neutrophils was corroborated *in vivo* as adoptive transfer of IL-10 deficient neutrophils into infected WT recipient failed to dowregulate IFN-γ production and to increase survival although they significantly controlled blood parasite numbers. Again, this result highlights that the solely reduction in parasitemia is not enough to prevent mortality in conditions of important tissue damage. Of note, IL-17RA deficient neutrophils produced similar amounts of IL-10 and, accordingly, showed the same suppressor ability than WT counterparts *in vitro*, suggesting that IL-17RA signaling in the neutrophils themselves is not required for the acquisition of the “regulatory” phenotype or migration into tissues. Toll-like receptor ligands [61] as well as the acute-phase reactant serum amyloid A [62] have been shown to promote differentiation of IL-10 producing neutrophils. As the *T. cruzi* bear several TLR-2 and -4 ligands [64] and promote serum amyloid A production by macrophages [65], it is likely that during this parasite infection both signals cooperate to induce a proper environment for the induction of suppressor IL-10 producing neutrophils.

Altogether, our results demonstrated that IL-17RA regulates IFN-γ production and tissue damage, at least in part, by recruiting regulatory IL-10-producing neutrophils during *T. cruzi* infection. Although the requirement of IL-10 to prevent inflammatory damage and mortality during *T. cruzi* infection was reported many years ago [32], [33], our results provide important new information about the sources of IL-10 and position neutrophils as an important regulatory cells expanding our understanding of the complex mechanisms that regulate inflammation during this parasite infection. Furthermore, to our knowledge, this is the first report linking IL-17-mediated protective effect during infections with the recruitment of IL-10 producing neutrophils and the consequent regulation of exacerbated type 1 inflammatory responses. IL-17 producing T cells were reported to be protective also in mycobacterial infection [66] where suppressive mechanisms mediated by IL-10 secreting neutrophils were able to temper pathological inflammatory responses but also to impair complete bacterial clearance [61]. Considering these antecedents and our results it is likely that the regulatory mechanism involving IL-17-mediated recruitment of suppressor neutrophils may be extrapolative to other infections where the balance between inflammation and regulation prevents excessive host tissue damage but also favors pathogen persistence and chronicity of the infection.

## Materials and Methods

### Ethic statement

All animal experiments were approved by and conducted in accordance with guidelines of the Committee for Animal Care and Use of the Facultad de Ciencias Químicas, Universidad Nacional de Córdoba (Approval Number HCD 274/09) in strict accordance with the recommendation of the Guide to the Care and Use of Experimental Animals published by the Canadian Council on Animal Care (OLAW Assurance number A5802-01).

### Mice

IL-17RA deficient mice were provided by Amgen Inc through Master Agreement N° 200716544. IL-10 deficient mice were purchased from The Jackson Laboratories (USA). C57BL/6 wild type mice were obtained from School of Veterinary, La Plata National University (La Plata, Argentina). All animals were housed in the Animal Facility of the Facultad de Ciencias Químicas, Universidad Nacional de Córdoba.

### Parasites and experimental infection

Mice used for experiments were sex- and age-matched, and housed with a 12-h light-dark cycle. Bloodstream trypomastigotes of the Tulahuén strain of *T. cruzi* were obtained from BALB/c mice infected 10 days earlier. Mice were inoculated intraperitoneally (i.p.) with 0.2 ml PBS containing 3×10^3^ trypomastigotes. Parasitemia was monitored by counting the number of viable trypomastigotes in blood collected from the retrorbital sinus or tail vein after lysis with a 0.87% ammonium chloride buffer. Mouse survival was followed every day.

### Cell preparation, sorting and culture

Spleen, liver and inguinal lymph nodes were obtained and homogenized through a tissue strainer. Erythrocytes in spleen and liver cell suspensions were lysed for 5 min in Tris-ammonium chloride buffer. Liver infiltrating cells were obtained after 20 min centrifugation (600 g) in a 35% and 70% bilayer Percoll (Sigma) gradient. Bone marrow cells were isolated by flushing femurs and tibias of mice with PBS-2%FBS. Viable cell numbers were determined by trypan blue exclusion using a Neubauer counting chamber. Cell subsets (NK cells, CD4 and CD8 T cells, neutrophils and the remaining negative fraction) from spleen and liver were purified to >98% by cell sorting after five color staining using the following antibodies: CD11b-FITC, CD4-PE, CD8-PECy7, CD3-PerCPCy5.5, NK1.1-Alexa Fluor 647 (eBioscience). Bone marrow neutrophils were isolated by positive selection with anti-Ly6G-biotin and anti-biotin beads following manufacturer's instructions (Miltenyi Biotec). Purity of magnetic-isolated neutrophils was higher than 95% as determined by flow cytometry using CD11b, Ly-6G and Gr-1 staining (Figure S6A in Text S1). Neutrophil viability was higher than 98% as determined by trypan blue exclusion. Neutrophils from spleen and liver were purified to >98% (Figure S6B in Text S1) by cell sorting after six-color staining using the following antibodies: CD11b-FITC, CD4-PE, CD3-PerCPCy5.5, B220-Alexa Fluor 647, Ly-6G-PE-Cy7, Gr-1-Alexa Fluor 750 (eBioscience). For sorting experiments, at least 3–5 mice were pooled by group. Cells were sorted with FACSAria (BD Bioscience). Cells were cultured in RPMI 1640 (Gibco, Invitrogen) medium supplemented with 2 mM glutamine (Gibco, Invitrogen), 50 µM 2-ME (Sigma), and 40 µg/ml gentamicin (Fabra Laboratories) containing 10% FBS (PAA).

### Cytokine and chemokine quantification

Cytokine-producing capacity of cell suspensions or purified subsets was assessed after stimulation at a cell density of 1×10^6^ cell/ml. Cell suspension from spleen, lymph nodes and liver were stimulated during 48 h with 2 µg/ml anti-CD3 Abs (BD Biosciences) and 50 nM phorbol-12-13-dibutyrate (PdBU) (Sigma). Alternatively, cells were stimulated with low doses PMA (10 nM) and Ionomycin (0,5 µg/ml). Neutrophils were stimulated during 48 h with 1 µg/ml Pam_3_CSK_4_ (Invivogen), and live trypomastigotes (MOI 1∶1). IFN-γ, TNF, IL-10, IL-1β, IL-6 and IL-17E concentrations in culture supernatants or in plasma were assessed by ELISA using paired Abs or specific kits (eBiosciences) according to standard protocols. IL-17A and IL17F concentrations in culture supernatants or in serum were assessed using the FlowCytomix Multiple Analyte Detection System (eBiosciences) following manufacturer's instruction. For chemokines and G-CSF quantification, tissues were homogenized in PBS containing 0,5%BSA, 0,4M NaCl, 1 mM EDTA, 0,05% Tween 20 and a protease inhibitor cocktail (Sigma-Aldrich) and centrifuged at 10000 g during 10 min (adapted from [67]). CXCL1 and G-CSF were quantified in the supernatants using the CBA Assay from BD Biosciences following manufacturer's instruction. CXCL2 (MIP-2) and CXCL10 (IP-10) were quantified using the specific ELISA kits from Peprotech following manufacturer's instruction. Chemokines and G-CSF concentrations were normalized to total protein concentration determined by Bradford's technique (Biorad) in tissue homogenates.

### Flow cytometry

Cell suspensions were washed in ice-cold FACS buffer (PBS-2% FBS) and incubated with fluorochrome labeled-Abs for 20 min at 4°C. Different combinations of the following Abs were used: FITC-labeled: anti- CD19 or CD11b, PE-labeled: anti- CD11c or CD4, PECy7-labeled: anti- Ly6G or CD8, PerCPCy5.5-labeled: anti- CD4, CD3 or Ly6C, APC or Alexa Fluor 647-labeled: F4/80, Alexa Fluor 750-labeled: anti Gr-1. Intracellular cytokines were detected after stimulating cells during 5 hours with 50 nM PMA and 0.5 µg/ml ionomycin (Sigma), 1 µg/ml Pam_3_CSK_4_ (Invivogen), and live trypomastigotes (MOI 1∶1) in the presence of GolgiStop (BD Biosciences). Cells were surface-stained, fixed and permeabilized with BD Cytofix/Cytoperm and Perm/Wash (BD Biosciences) according manufacturer's instruction. Cells were incubated with APC-labeled antibody to IFN-γ, IL-17F or TNF and PE-labeled antibody to IL-17A, IL-6, IL-12p70 or IL-10 (BD Biosciences). Cells were acquired on FACSCanto II (BD Bioscience).

### Liver histology and transaminase activity

Livers obtained from WT and IL-17RA KO mice at different times post *T. cruzi* infection were fixed in formaldehyde and embedded in paraffin. Five-µm thick sections were examined by light microscopy after hematoxylin/eosin staining. Double-blind evaluation of liver damage focused in three main aspects: presence and type of inflammatory infiltrate, presence and extension of necrotic areas and hyaline degeneration and presence of cellular alterations such as vacuolization, swelling, nuclear alterations, etc. Photographs were taken using a Nikon Eclipse TE 2000 U equipped with a digital video camera. Plasma aspartate aminotransferase (AST) and alanine aminotransferase (ALT) activities were measured using commercial kits (Wiener Lab) following manufacturer's instruction.

### Cytospin of purified neutrophils

Two×105 sorted neutrophils were washed twice and dilute in 100 µl of ice-cold 2% FBS-PBS. Samples were aliquoted in the appropriate cytospin clips and spun in a cytocentrifuge at medium speed for 5–6 minutes. Slides were dried overnight, fixed with methanol and stained with May-Grünwald/Giemsa following a standard protocol.

### Treatment with neutralizing or depleting Abs

IFN-γ was neutralized *in vivo* by intraperitoneal injection of 250 µg of rat anti–mouse IFN-γ mAb (clone R4-6A2) at days 9, 12, 15 and 18 post-infection. The delayed kinetic of neutralization was chosen to allow an initial IFN-γ response required to avoid uncontrolled parasite spreading and early mortality. R4-6A2 mAb was prepared from the eponymous cell clone and purified by affinity chromatography using HiTrap Protein G columns (GE Healthcare). As control, mice were injected with equal quantities of normal rat IgG (Jackson ImmunoResearch). Mice were sacrificed at day 20 post-infection. Neutrophil depletion was achieved by intravenous injection of 250 µg rat anti-mouse Ly6G (clone 1A8, BioXCell) mAb or normal rat IgG (Jackson ImmunoResearch) as control at days 9, 12, 15 and 18 post-infection. Mice were sacrificed at day 20 post-infection unless used for survival monitoring. Neutrophil depletion efficiency was evaluated by determining the presence of CD11b+Gr-1+Ly-6G+ cells by flow cytometry.

### In vitro assessment of neutrophil regulatory activity

Splenocytes from normal WT mice were labeled with 0.5 µM CFSE (carboxyfluorescein succinimidyl ester, Invitrogen). Spleen CD11b+Ly-6G+ neutrophils were sorted from 20-day *T. cruzi* infected WT and IL-17RA KO mice. CFSE stained splenocytes (5×10^5^) and sorted neutrophils (1×10^5^) were cultured during 5 days in anti-CD3/anti-CD28 Ab coated (2 µg/ml each) 96-well plates in the presence or absence of 10 µg/ml of a blocking anti-IL-10R Ab (BD Biosciences). After culture, CFSE dilution and intracellular IFN-γ expression within de CD3 positive population were assessed by flow cytometry. Proliferation data are presented as relative to the percentage of CFSE-low CD3+ splenocytes in the absence of neutrophils and anti-IL-10R Ab (set as 100% proliferation).

### Neutrophil adoptive transfer

To assess neutrophil recruitment to different tissues, bone marrow neutrophils from WT were stained with CFSE and bone marrow neutrophils from IL-17RA KO mice were stained with SNARF-1 (Molecular Probes, Invitrogen) following conventional protocols prior to injection in the retrorbital sinus of non-infected and 20-day *T. cruzi*-infected WT and IL-17RA KO mice. Mice were sacrificed 3 h after injection of 5×10^6^ CFSE-stained WT neutrophils and of 5×10^6^ SNARF-stained IL-17RA KO neutrophils and the presence of transferred neutrophils were evaluated in blood, bone marrow, liver and spleen. To evaluate the effect of neutrophils adoptive transfer during *T. cruzi* infection, WT or IL-17RA KO mice received four intravenous injections with a total of 5×10^6^ purified bone marrow WT neutrophils in 0,2 ml PBS at days 9, 12, 15 and 18 post-infection. Injections were performed in the retrorbital sinus alternating the eyes. In those adoptive transfer experiment using WT and IL-10 KO neutrophils, 2×10^6^ neutrophils were injected. Control mice received 0,2 ml PBS. Mice were sacrificed at day 20 post-infection unless used for survival monitoring.

### Statistics

Statistical significance of comparisons of mean values was assessed by a two-tailed Student's t test, two-way ANOVA followed by Bonferroni's posttest and a Gehan-Breslow-Wilcoxon test using GraphPad software.

### Accession numbers

B220/Ptprc (NM_001111316.1); CD11b/Integrin alpha M (NM_001082960.1); CD11c/Integrin alpha X (NM_021334.2); CD28 (NM_007642.4); CD3d (NM_013487.3); CD3e (NM_007648.4); CD3g (NM_009850.2); CD4 (NM_013488.2); CD8a (NM_001081110.2); CD8b.1 (NM_009858.2); CXCL1 (NM_008176.3); CXCL10 (NM_021274.2); CXCL2 (NM_009140.2); G-CSF (NM_009971.1); GM-CSF (NM_009969.4); IFNg (NM_008337.3); IL10 (NM_010548.2); IL10RA (NM_008348.2); IL12a (NM_001159424.1); IL12b.1 (NM_008353.2); IL17A (NM_010552.3); IL17B (NM_019508.1); IL17C (NM_145834.3); IL17D (NM_145837.3); IL17F (NM_145856.2); IL17RA (NM_008359.2); IL17RB (NM_019583.3); IL17RC (NM_178942.1); IL17RD (NM_134437.3); IL17RE (NM_145826.5); IL1b (NM_008361.3); IL25/IL17E (NM_080729.3); IL27Ra (NM_016671.3); IL6 (NM_031168); Ly6c1 (NM_010741.3); Ly6G (XM_001475753.2/XM_909927.3); NKRP1C (NM_001159904.1); TGFb.1 (NM_011577.1); TNF (NM_013693.2).

## Supporting Information

Text S1 Six supporting figures are available in Text S1. **Figure S1. Time course histological evaluation of hepatic damage during** ***T. cruzi*** **infection.** Photographs (200×) of Hematoxilin/Eosin stained liver sections from WT and IL-17RA KO mice non-infected (NI) or after different times post *T. cruzi* infection. Head arrows indicate focal inflammatory infiltrates. Black lines delineate extensive necrotic areas. Stars indicated hyaline degeneration. The analysis of the micrographs is summarized in Table 1. Photographs are representative of one out of five mice. Data is representative of two independent experiments. **Figure S2. Increased production of IFN-γ in** ***T. cruzi*** **infected IL-17RA KO mice.** A) Expression of IFN-γ and CD3 after 5 h PMA/Ionomycin stimulation of spleen and liver cell suspensions obtained from IL-17RA KO and WT mice after 20 days of *T. cruzi*-infection. Plot representative of one out of five mice. B) Concentration of IFN-γ (left) and TNF (right) detected in the supernantants of CD4+ and CD8+ T cells sorted from spleen (top) and liver (bottom) of 20-day *T. cruzi* infected WT and IL-17R KO mice and cultured for 48 h with anti-CD3 and PDBu. Data are shown as mean ± SD of triplicate cultures. P values calculated with two-tailed T test. Data are representative of three independent experiments. **Figure S3. Phenotype and morphology characterization of neutrophils infiltrating liver and spleen during** ***T. cruzi*** **infection.** A) Expression of Ly-6G, Ly-6C, CD11c and F4/80 in the CD11b+Gr-1+ cells from spleen and liver of 20-day *T. cruzi* infected WT and IL-17RA KO mice. Dot plots and histograms are representative one out of five mice per group. B) Morphology of the CD11b+Gr-1+ cells from spleen and liver of 20- *T. cruzi* infected WT and IL-17RA KO mice. Data in A–B are representative of two independent experiments. **Figure S4. Similar frequencies of CD11b+Gr-1+ cell population in bone marrow and blood of infected WT and IL-17RA KO mice.** Percentage (A) and absolute numbers (B) of CD11b+Gr-1hi neutrophils in the bone marrow (left panels) and CD11b+Gr-1+ cells in the blood (right panels) of WT and IL-17RA KO mice non-infected (NI) or after 20 days of *T. cruzi* infection. Dot plots are representative one out of five mice per group. C) Concentration of G-CSF in spleen and liver homogenates from non-infected (NI) or 20-day infected (I) WT and IL-17RA KO mice. Data are shown as mean ± SD of biological triplicates, n = 5 mice per group and normalized to total protein concentration. P values calculated with two-tailed T test. Data in A–B and in C are representative of four and two independent experiments, respectively. **Figure S5. Cytokine production by bone marrow neutrophils stimulated with live** ***T. cruzi*** **.** Percentage of IL-10 (A), TNF and IL-6 (B) and IL-12p70 (C) producing cells after 6 h stimulation with live *T. cruzi* and Pam3CSK4 of Ly-6G+ neutrophils purified from bone marrow of WT and IL-17RA KO mice. Plots are representative of triplicate cultures. Data are representative of two independent experiments. **Figure S6. Purity of neutrophils after magnetic isolation and cell sorting.** A) Purity of neutrophils isolated from bone marrow of WT mice by magnetic positive selection as determined by CD11b, Ly-6G and Gr-1 staining. B) Purity of neutrophils isolated from spleen of WT mice by cell sorting as determined by CD11b, Ly-6G and Gr-1 staining. Plots are representative of all the purification experiments.
(PDF)
