# Supplementary figures and images for: IL-17RA Signaling Reduces Inflammation and Mortality during Trypanosoma cruzi Infection by Recruiting Suppressive IL-10-Producing Neutrophils
Source: PLoS Pathog. 2012 Apr 26;8(4):e1002658. doi: 10.1371/journal.ppat.1002658 (PMC3343119; doi:10.1371/journal.ppat.1002658)

Figure S1

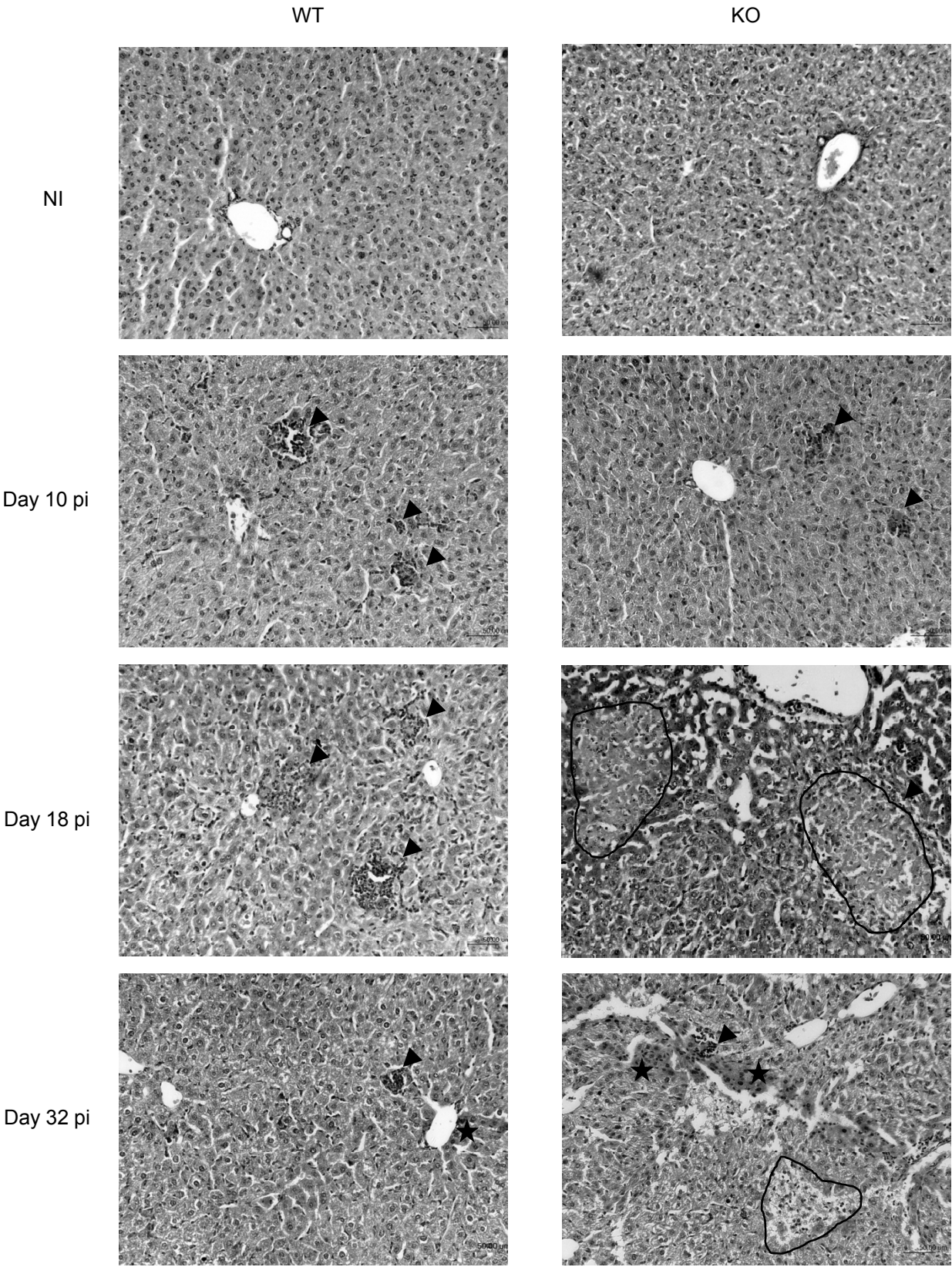

Figure S2

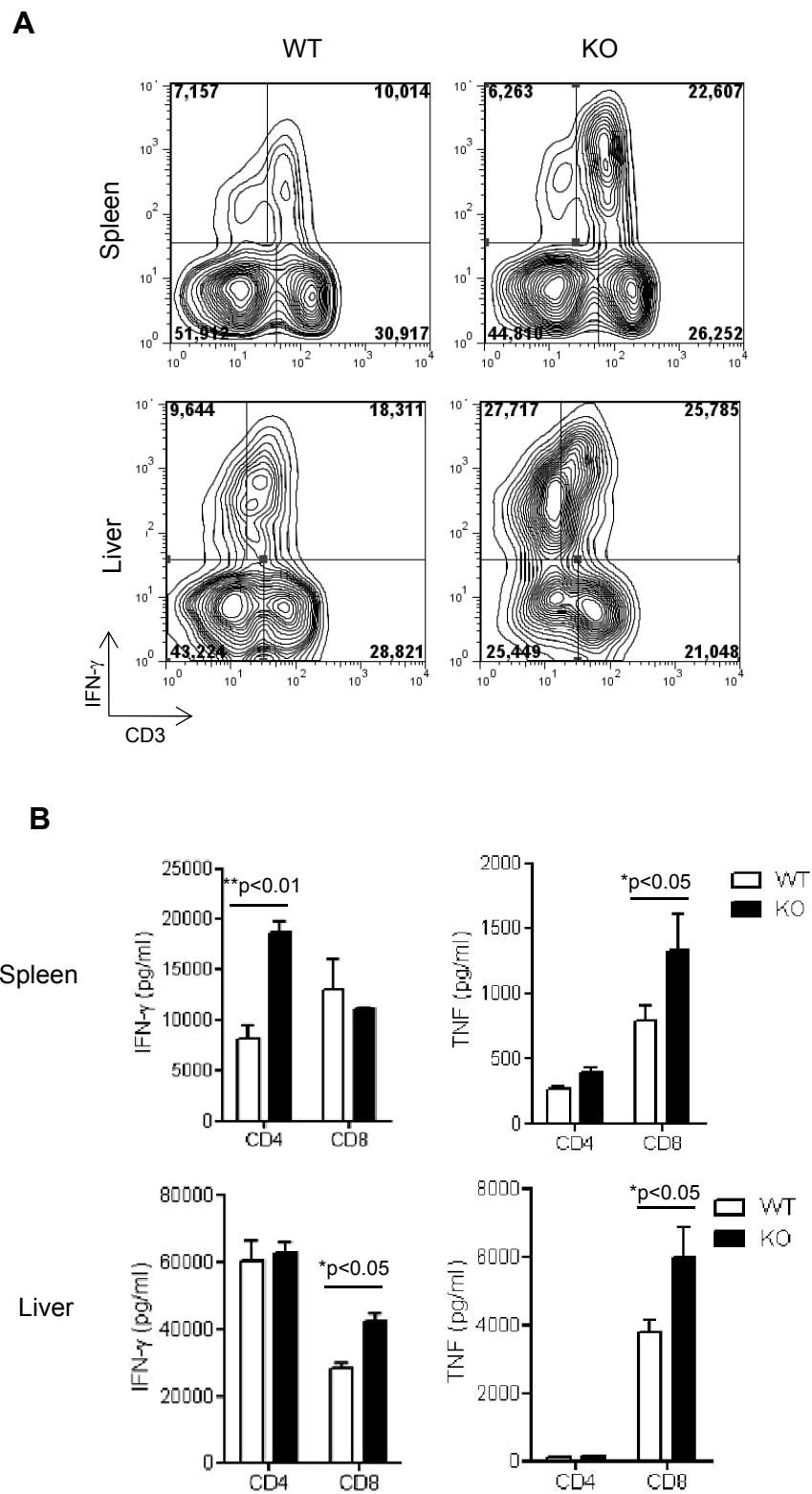

**Figure S3**

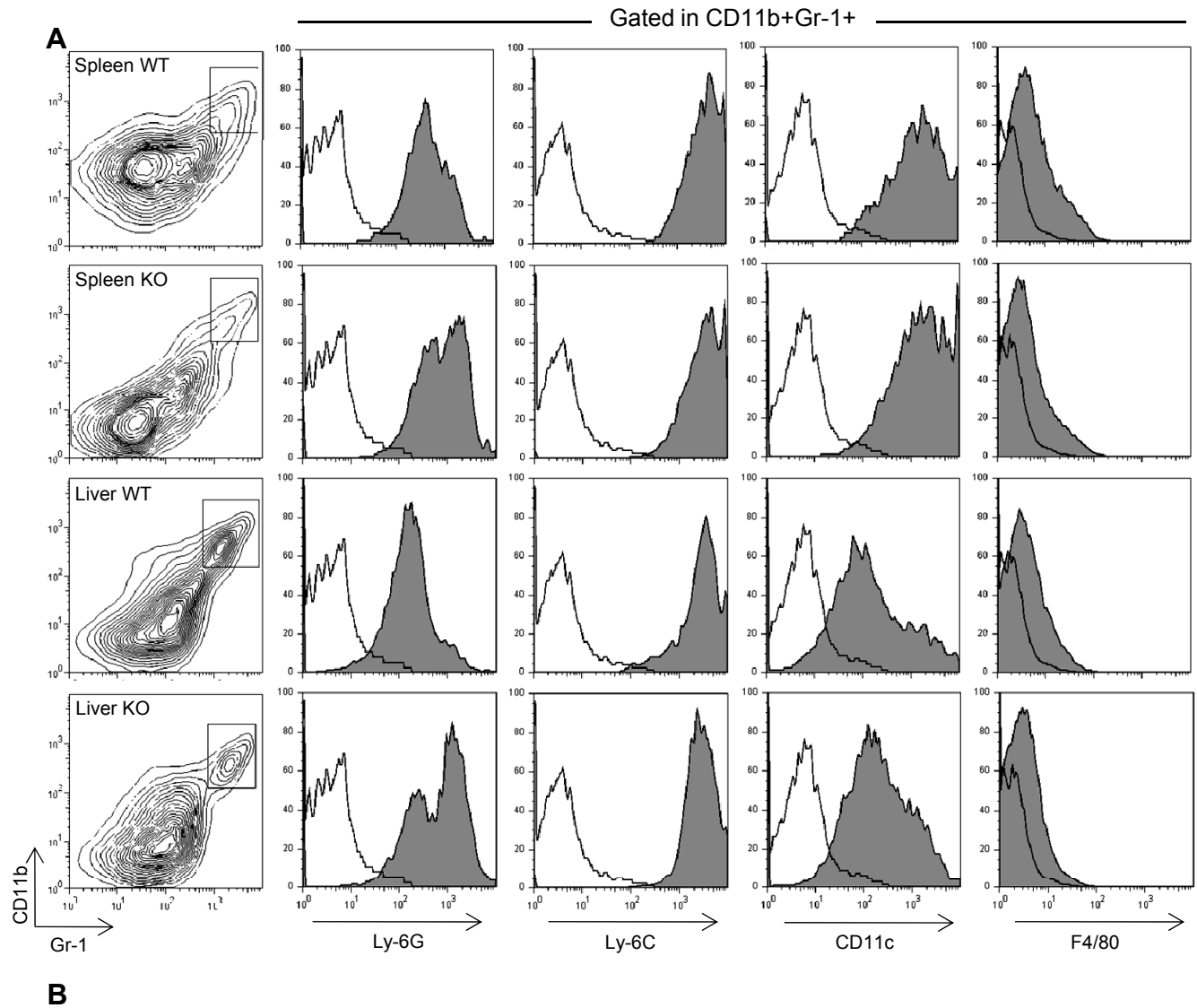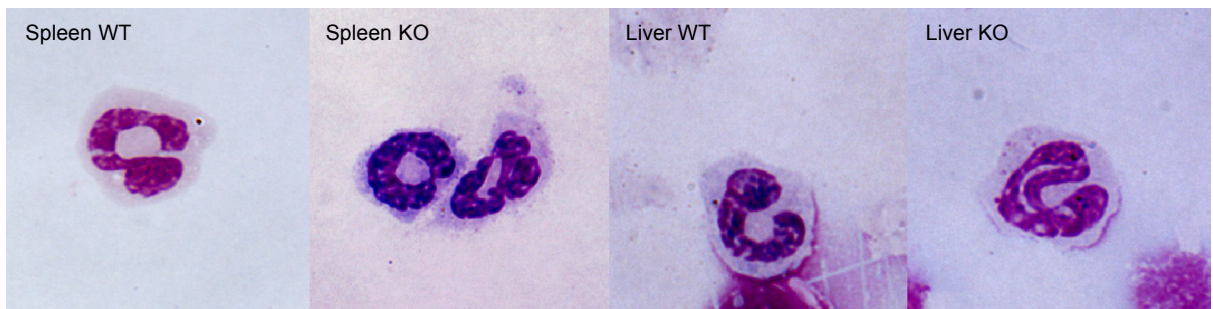

Figure S4

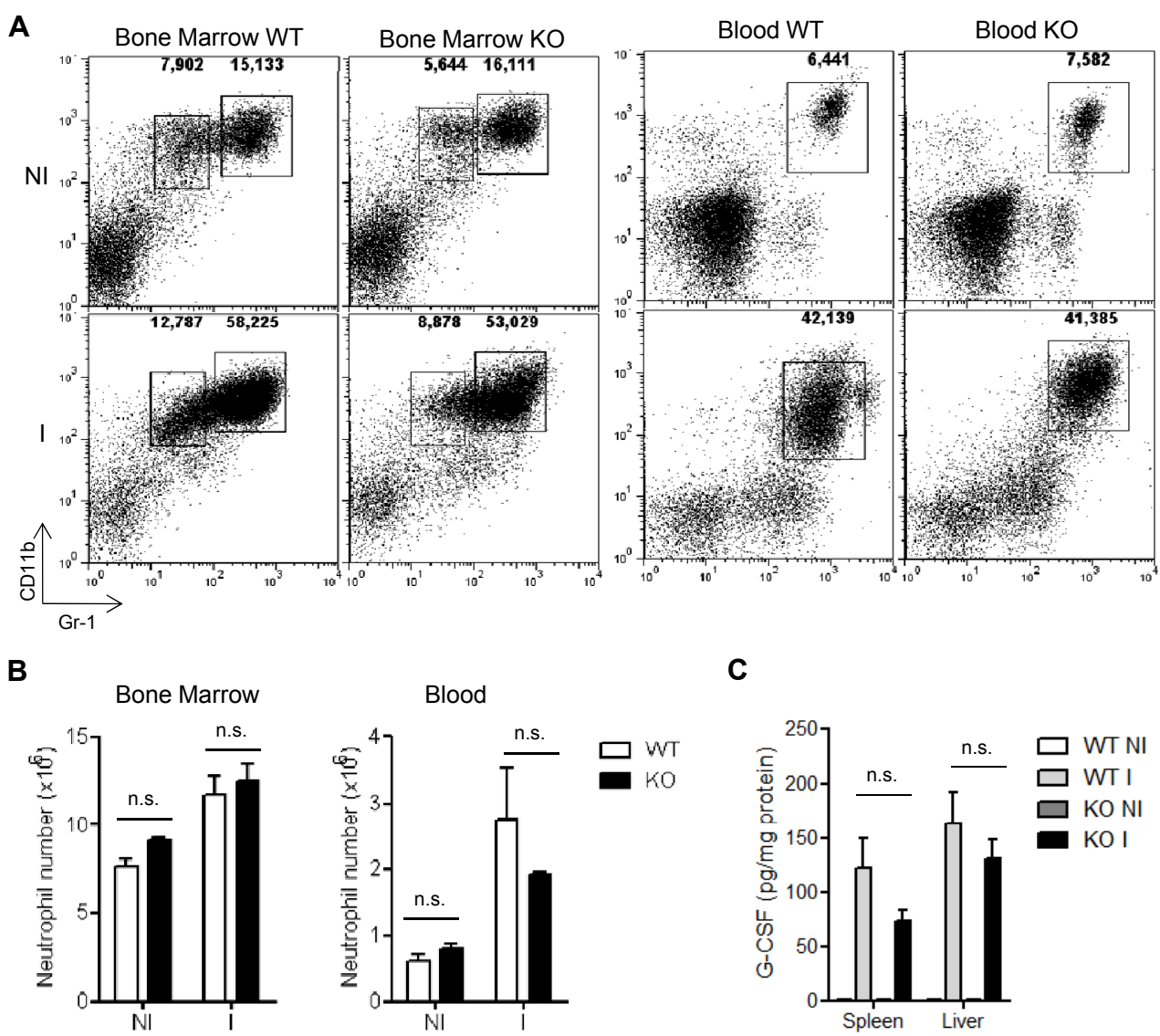

Figure S5

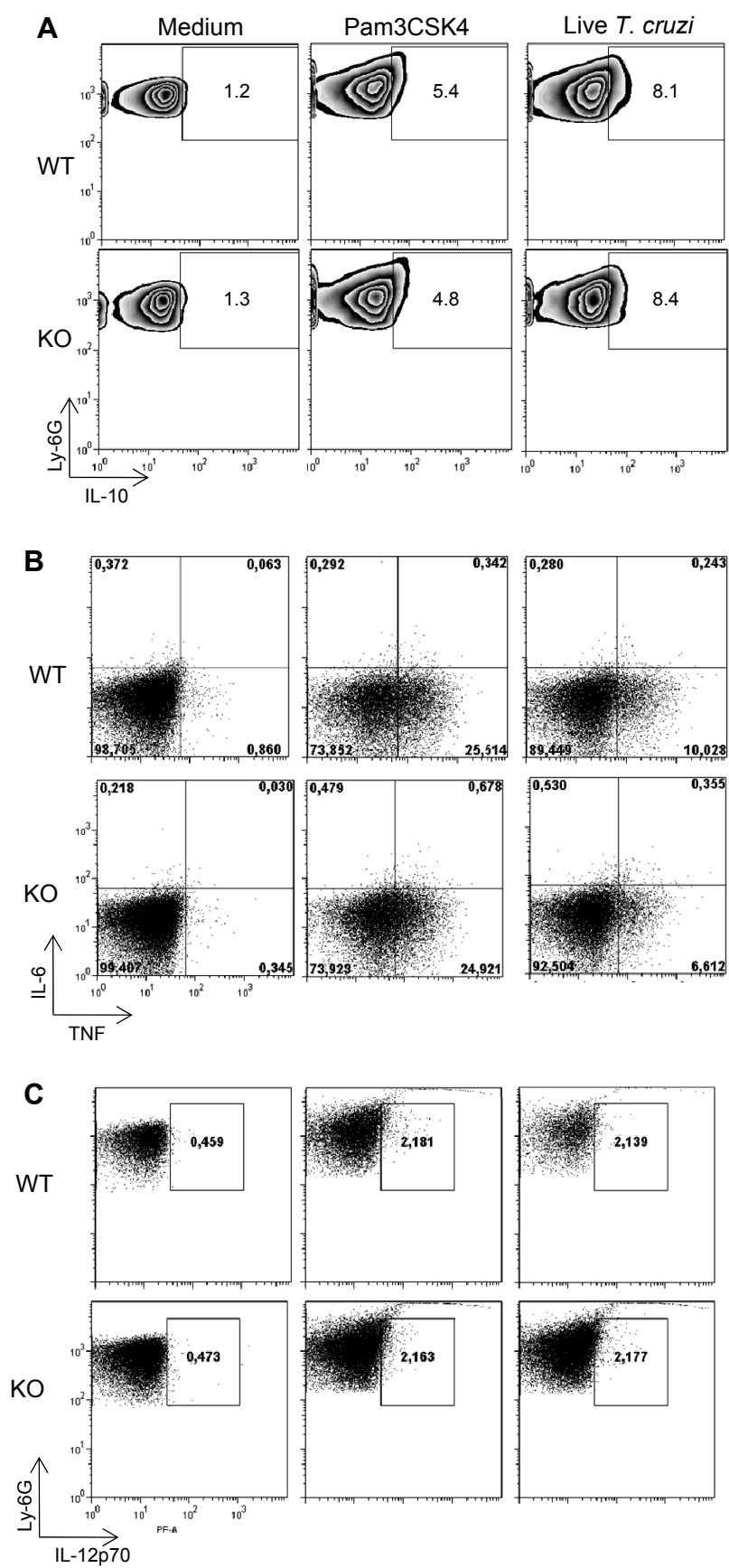

Figure S6

A

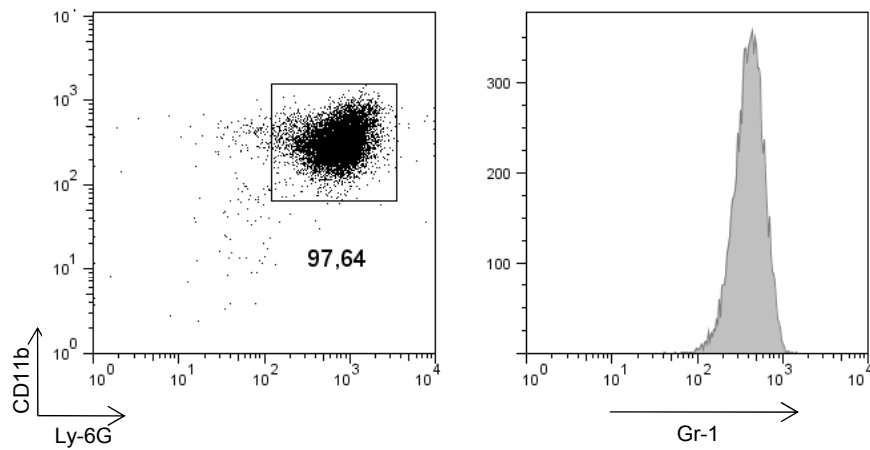

B

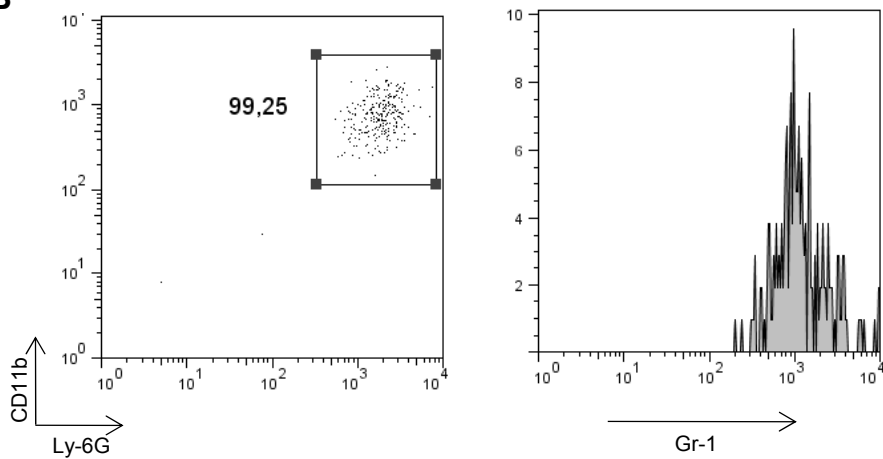

Supplement: Text S1 — Six supporting figures are available in Text S1. Figure S1. Time course histological evaluation of hepatic damage during T. cruzi infection. Photographs (200×) of Hematoxilin/Eosin stained liver sections from WT and IL-17RA KO mice non-infected (NI) or after different times post T. cruzi infection. Head arrows indicate focal inflammatory infiltrates. Black lines delineate extensive necrotic areas. Stars indicated hyaline degeneration. The analysis of the micrographs is summarized in Table 1. Photographs are representative of one out of five mice. Data is representative of two independent experiments. Figure S2. Increased production of IFN-γ in T. cruzi infected IL-17RA KO mice. A) Expression of IFN-γ and CD3 after 5 h PMA/Ionomycin stimulation of spleen and liver cell suspensions obtained from IL-17RA KO and WT mice after 20 days of T. cruzi-infection. Plot representative of one out of five mice. B) Concentration of IFN-γ (left) and TNF (right) detected in the supernantants of CD4+ and CD8+ T cells sorted from spleen (top) and liver (bottom) of 20-day T. cruzi infected WT and IL-17R KO mice and cultured for 48 h with anti-CD3 and PDBu. Data are shown as mean ± SD of triplicate cultures. P values calculated with two-tailed T test. Data are representative of three independent experiments. Figure S3. Phenotype and morphology characterization of neutrophils infiltrating liver and spleen during T. cruzi infection. A) Expression of Ly-6G, Ly-6C, CD11c and F4/80 in the CD11b+Gr-1+ cells from spleen and liver of 20-day T. cruzi infected WT and IL-17RA KO mice. Dot plots and histograms are representative one out of five mice per group. B) Morphology of the CD11b+Gr-1+ cells from spleen and liver of 20- T. cruzi infected WT and IL-17RA KO mice. Data in A–B are representative of two independent experiments. Figure S4. Similar frequencies of CD11b+Gr-1+ cell population in bone marrow and blood of infected WT and IL-17RA KO mice. Percentage (A) and absolute numbers (B) of CD11b+ [file ppat.1002658.s001.pdf]
